# Development of a Multi-Locus Real-Time PCR with a High-Resolution Melting Assay to Differentiate Wild-Type, Asian Recombinant, and Vaccine Strains of Lumpy Skin Disease Virus

**DOI:** 10.3390/vetsci12030213

**Published:** 2025-03-01

**Authors:** Kultyarat Bhakha, Yuto Matsui, Natchaya Buakhao, Saruda Wanganurakkul, Taweewat Deemagarn, Mami Oba, Hitoshi Takemae, Tetsuya Mizutani, Naoaki Misawa, Lerdchai Chintapitaksakul, Kentaro Yamada, Nutthakarn Suwankitwat

**Affiliations:** 1 Virology Laboratory, National Institute of Animal Health, Department of Livestock Development, Bangkok 10900, Thailand; kultyarat.b@dld.go.th (K.B.); natchaya.anb@gmail.com (N.B.); lerdchai@gmail.com (L.C.); 2 Center for Animal Disease Control, University of Miyazaki, 1-1 Gakuenkibanadai-nishi, Miyazaki City, Miyazaki 889-2192, Japan; matsui.yuto.g4@cc.miyazaki-u.ac.jp (Y.M.); a0d901u@cc.miyazaki-u.ac.jp (N.M.); 3 Veterinary Research and Development Center (Eastern Region), Department of Livestock Development, Chonburi 20220, Thailand; aunsarudaku61@gmail.com; 4 Animal Health Research and Innovation Promotion Section, National Institute of Animal Health, Department of Livestock Development, Bangkok 10900, Thailand; deemagarn@hotmail.com; 5 Center for Infectious Diseases of Epidemiology and Prevention Research (CEPiR), Tokyo University of Agriculture and Technology, Fuchu City, Tokyo 183-8509, Japan; ft4018@go.tuat.ac.jp (M.O.); fy7210@go.tuat.ac.jp (H.T.); tmizutan@cc.tuat.ac.jp (T.M.); 6 Laboratory Veterinary Public Health, Department of Veterinary Sciences, Faculty of Agriculture, University of Miyazaki, 1-1 Gakuenkibanadai-nishi, Miyazaki City, Miyazaki 889-2192, Japan

**Keywords:** lumpy skin disease virus, DIVA, real-time PCR, high-resolution melting analysis, multi-locus

## Abstract

Lumpy skin disease virus (LSDV) affects cattle and causes significant economic damage. The live vaccine derived from an attenuated strain is effective but is associated with mild disease and skin lesions in some vaccinated cattle. Moreover, recombinant LSDV strains, particularly one with wild-type field and vaccine strains, have recently emerged and spread throughout Asian countries. A cost-effective LSDV typing method is required. We developed a multi-locus real-time PCR with a high-resolution melting (HRM) assay to differentiate between the wild-type, vaccine, and dominant Asian recombinant strains. Based on a multiple alignment analysis, we selected three target genes for the HRM assay, ORF095, ORF126, and ORF145, in which there are insertions/deletions and nucleotide substitutions between wild-type and vaccine strains, and designed primer sets for the assay. Using the synthetic DNA encoding these genes for the two strains, it was shown that the PCR amplicons intercalated with a saturating fluorescent dye could clearly differentiate between wild-type and vaccine strains in the HRM analysis for all three target genes. Further, using clinical samples, our method was able to identify recombinant strains harboring the wild-type ORF095 and ORF145 and the vaccine strain ORF126 genes. Thus, our HRM assay may provide rapid LSDV typing.

## Simple Summary

Lumpy skin disease (LSD) is a transboundary viral disease of cattle, the clinical signs of which are fever, anorexia, skin nodules, decreased milk production, infertility, and abortion, leading to severe economic losses. A live-attenuated vaccine is available to control this disease, but a strategy for the differentiation of infected from vaccinated animals (DIVA) is needed because of the side effects of the vaccine. Recently, a recombinant strain, which is a hybrid of the wild-type field and vaccine strains, has spread throughout Asian countries. Therefore, a rapid and low-cost method to differentiate field, vaccine, and recombinant strains is needed. We chose the high-resolution melting (HRM) assay, which is a post-PCR assay capable of discriminating gene variations without sequencing, and selected three genes (ORF095, ORF126, and ORF145) to establish the differentiation assay. In this study, we found that each gene can be distinguished as the field or vaccine type by each HRM assay, and the multi-locus HRM assay can identify the recombinant viruses that have the wild-type ORF095 and ORF145 genes and the vaccine-type ORF126 gene within 2 h of the PCR run. We believe that this method will be useful to control LSD in many countries.

## 1. Introduction

Lumpy skin disease (LSD) is a transboundary viral disease affecting domestic and wild ruminants, especially cattle and water buffalo. Several clinical signs such as fever, anorexia, skin nodules, enlarged lymph nodes, decreased milk production, infertility, and abortion often appear in cattle affected by LSD. Although the mortality rate is low (1–5%), LSD is listed as a notifiable disease by the World Organization for Animal Health (WOAH) because of its significant economic impact on the livestock industry, notably reduced commercial value [1,2,3]. LSD is caused by lumpy skin disease virus (LSDV)—a novel taxonomic name is *Capripoxvirus lumpyskinpox*—which is an enveloped virus belonging to the genus *Capripoxvirus* of the family Poxviridae. The sheeppox and goatpox viruses, SPPV and GTPV—their novel names are *C. sheeppox* and *C. goatpox*—are in the same genus as LSDV, and the genome sequences of these two viruses are 97% identical to LSDV [4]. Because these viruses have only one serotype, they are difficult to distinguish by serological or antigenic methods [5,6]. The genome of LSDV comprises double-stranded DNA of 151 kbp containing 156 open reading frames (ORFs) [7]. LSDV is shed in bodily fluids such as eye, nose, and mouth secretions and semen. Contact with these fluids can be a source of LSDV infection [8,9], but the primary route of infection is thought to be mechanical transmission by various arthropods [10,11,12].

LSD was first reported in Zambia as pseudo-urticaria in 1929. Then, LSD spread widely in African countries, and the Neethling strain of LSDV, which is the parent strain of the current live-attenuated vaccine strains, was isolated in South Africa in 1957 [13]. The first confirmed outbreak outside of Africa was in Israel in 1989, spreading to Middle Eastern countries by 1990 and to the Balkans and Russia by 2015 [13,14]. In 2017, the vaccine-like strain LSDV RUSSIA/Saratov/2017 was discovered in Saratov, a region of the Russian Federation bordering Kazakhstan. Full-length nucleotide sequence analysis revealed that the strain’s genome contains a patchwork of wild-type field strain DNA that previously caused outbreaks on the DNA backbone of a live-attenuated LSD vaccine strain [15,16]. Such a vaccine-like strain is called a recombinant strain, and several recombinant strains have been identified since the emergence of LSDV RUSSIA/Saratov/2017. Whole-genome sequencing of these recombinant strains revealed differences in recombination events and SNPs, which prompted division into five clusters, tentatively named 2.1–2.5 [13]. In particular, the recombinant strains in cluster 2.5 spread extensively throughout East and Southeast Asia [17,18,19,20,21,22,23]. In Thailand, the first outbreak of the recombinant strains was reported in Roi Et in March 2021 [24]. Meanwhile, LSD outbreaks caused by the wild-type field strains of Kenyan origin were also reported from India to Myanmar [25,26,27,28,29,30,31]. Because Myanmar and Thailand share a border, there is concern that the wild-type field strain may be introduced into Thailand in the future. Therefore, a rapid diagnostic method is needed that can distinguish between an outbreak caused by an existing recombinant strain and one caused by an invading wild-type field strain.

A live-attenuated vaccine against LSDV (Neethling-LW-1959) was developed in the 1960s in South Africa by attenuating the Neethling-type field strain (Neethling/1957 isolate) of LSDV [13,32]. Although the homologous vaccine provides good protection against virulent field strains for cattle, it has been reported to cause side effects of localized skin reactions at the vaccination site, generalized small skin nodules, and a temporary decrease in milk production when cows are first immunized. These effects are known as the Neethling response [33,34,35,36,37]. Two live-attenuated vaccines, Lumpyvax (MSD, Pretoria, South Africa) and MEVAC (Kemin, Cairo, Egypt), have been used to prevent and control LSD in Thailand since June 2021 [24]. Therefore, there is a need for a rapid and low-cost method that can determine whether LSD clinical signs are caused by the vaccine strains (Neethling response) or by wild-type field or recombinant strains. A common method for identifying viral strains is genome sequencing after virus isolation using cultured cells from clinical specimens, but this is time-consuming and labor-intensive [17].

High-resolution melting (HRM) analysis is a method used to detect genetic diversity after real-time PCR using a fluorescent intercalating dye based on the different melting temperatures (Tm) of DNA amplicons. Although HRM analysis cannot provide specific sequence information, it can detect genetic differences in target genes—such as single nucleotide substitutions and deletions/insertions—cheaply and quickly and can be used for human single nucleotide polymorphism (SNP) analysis and pathogen strain classification [38,39,40,41,42,43,44,45,46]. Several classifications of LSDV strains by HRM analysis have been reported [47,48,49,50,51,52]. However, there is still no HRM assay that can distinguish between wild-type field strains, vaccine strains, and the recombinant strains widely prevalent in Asia. In the present study, we selected and validated three genes (ORF095, ORF126, and ORF145) for multi-locus real-time PCR with an HRM assay that can identify specific LSDV strains.

## 2. Materials and Methods

### 2.1. Plasmid and Viral DNA

The pUCFa plasmid vectors encoding ORF095, ORF126, and ORF145 of the vaccine strain SIS-Lumpyvax (GenBank No. KX764643) or the wild-type field strain LSDV-WB/IND/19 (GenBank No. OP297402), linked in tandem, were purchased from FASMAC (Kanagawa, Japan). The recombinant strain LSDV/Thailand/Yasothon/2021 (GenBank No. OM033705) and 15 skin nodule samples from Chainat, Chumphon, Lopburi, Bangkok, Ubon Ratchathani, Surin, Nan, and Chiang Mai provinces were collected by the National Institute of Animal Health (NIAH), Thailand. The wild-type field strain LSDV/210LSD-249/BUL/16 (GenBank No. MT643825), isolated in Bulgaria in 2106 [53], was provided by Dr. Nick De Regge of Sciensano in Belgium, the WOAH Reference Laboratory for LSD. The commercial vaccine MEVAC was also used for validation. Viral DNA was extracted and purified from samples using the High Pure PCR Template Preparation Kit (Roche Diagnostics, Mannheim, Germany) according to the manufacturer’s instructions.

### 2.2. Screening of LSDV Samples

TaqMan real-time PCR targeting the P32 gene of capripoxvirus was conducted to screen for the LSD-positive cases according to the WOAH recommendations. The reaction mixture comprised 20 μL, containing primers (200 nM each), a TaqMan minor groove binder (MGB) probe (120 nM), 5 μL of DNA template, and 10 μL of FastStart Essential DNA Probes Master (Roche, Basel, Switzerland). The primers and probes for the screening were as follows: CaPV-074F1, 5′-AAA ACG GTA TAT GGA ATA GAG TTG GAA-3′; CaPV-074R1, 5′-AAA TGA AAC CAA TGG ATG GGA TA-3′; and CaPV-074P1, 5′-FAM-TGG CTC ATA GAT TTC CT-MGB-NFQ-3′ [53]. A QuantStudio 5 Real-time PCR System (Applied Biosystems, Thermo Fisher Scientific, Waltham, MA, USA) was used for real-time PCR with the following conditions: 95 °C for 10 min and then 40 cycles of amplification (95 °C for 15 s and 60 °C for 45 s). The LSDV DNA levels were expressed as threshold cycle (Ct) values, which were determined using the default setting.

### 2.3. Primer Design for HRM Analysis

Nucleotide sequence data were analyzed using GENETYX Network Version 20.0.2 (Nihon Server Corperation, Tokyo, Japan). The whole-genome sequences of LSDV were aligned using Clustal W, which is embedded in the GENETYX software package, with default parameters. Based on the multiple alignment analysis (Figure S1), we selected three target genes (ORF095, ORF126, and ORF145). The primer sets used for real-time PCR with the HRM assay for LSDV classification were designed to bind to highly conserved regions flanking deletions or insertions of the target genes, with optimization using OligoAnalyzer (https://eu.idtdna.com/calc/analyzer, accessed on 29 June 2023). The sequences of the primer sets are listed in Table 1, and the primer set for ORF126 was reported previously [50].

### 2.4. Optimization of Real-Time PCR with the HRM Assay

The real-time PCR and HRM analysis was performed using the QuantStudio 5 Real-time PCR system and MeltDoctor HRM Master Mix (Applied Biosystems, Thermo Fisher Scientific Waltham, MA, USA) according to the manufacturer’s instructions with the following slight modifications: the reaction mixture was adjusted to a total volume of 20 μL, and the template DNA was adjusted to 2 μL. Primer concentrations of 125, 250, and 500 nM were evaluated for optimization using the plasmid DNAs described above; the optimized primer concentrations of ORF095, ORF126, and ORF145 were found to be 250, 125, and 500 nM, respectively. The cycling program was 95 °C for 10 min followed by 40 cycles of 95 °C for 15 s and 60 °C for 1 min. The melting curve stage ranged from 60 °C to 95 °C with a dissociation of 0.025 °C/s. The assay was completed within 2 h/run. The results were analyzed with High-Resolution Melt software V3.2 (Applied Biosystems, Thermo Fisher Scientific).

### 2.5. Determination of the Analytical Sensitivity and Specificity of the Assay

The analytical sensitivities with limits of detection (LODs) of the three primer pairs were determined using 10-fold serial dilutions ranging from 10^1^ to 10^9^ copies/μL of the plasmid DNA encoding the three genes of the wild-type field or vaccine strains described above. The experiment comprised three replicates at each dilution, plus negative and non-template controls. The LOD was defined as the lowest concentration at which a fluorescent signal could be detected in three wells per run. For the specificity test, nucleic acids derived from bovine viruses, i.e., pseudocowpox virus, bluetongue virus, infectious bovine rhinotracheitis virus, and bovine papillomaviruses, were selected and used to validate the HRM assay.

### 2.6. Statistical Analysis

Tm values were analyzed via Student’s *t*-test using GraphPad Prism version 8 (GraphPad Software, La Jolla, CA, USA), where a *p* value less than 0.05 was considered to indicate a significant difference.

## 3. Results

### 3.1. Determination of Target Genes for HRM Analysis to Classify LSDV Strains

Multiple alignment analysis of the whole-genome sequences of capripoxviruses revealed that there are 48 and 27 bp deletions in the ORF095 and ORF126 genes, respectively, and a 72 bp insertion and several nucleotide substitutions in the ORF145 gene in LSDV vaccine strains (Figure S1a–c). It was expected that large deletions would produce significant changes in Tm values. In addition, we found that Asian LSDV recombinant strains possess an ORF126 that is identical to the vaccine strain-derived sequence and ORF095 and ORF145 sequences that are identical to the wild-type field strain-derived sequences (Figure S1a–c). Therefore, we decided to combine the results of the HRM analysis for these three genes to classify LSDV strains. For example, we could identify a suspected LSD case to be the recombinant strain if the HRM results indicated that the ORF095 and ORF145 were wild-type field genes but ORF126 was the vaccine gene.

### 3.2. Evaluating the HRM Results of Three Genes for LSDV Differentiation

As described in the Materials and Methods, we designed primer sets for LSDV typing via HRM analysis that bind to the conserved regions flanking deletions/insertions in the ORF095 and ORF145 genes. The published primer set for the ORF126 gene [50], which binds upstream and downstream of the insertions detected in vaccine strains, was also used. Amplicon sizes from these primer sets were expected to be significantly different between the wild-type field and vaccine strains (Table 1).

**Figure 1:**
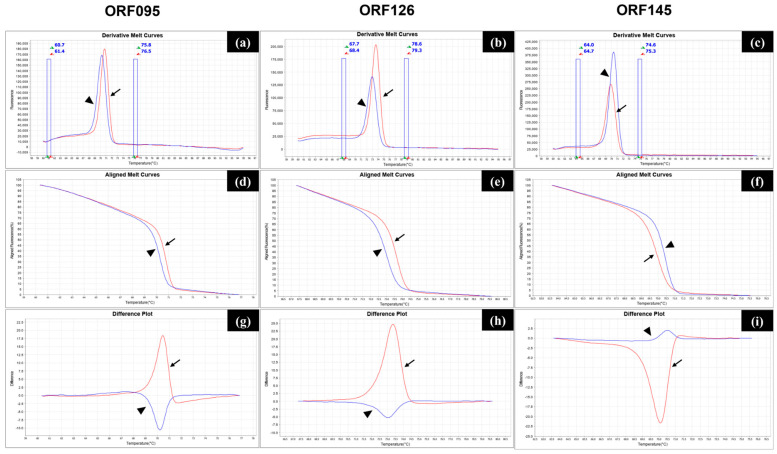
Evaluation of the high-resolution melt (HRM) analysis of target genes for differentiation using positive control plasmids. Each real-time PCR for the ORF095, ORF126, and ORF145 genes was performed on the QuantStudio 5 system using plasmids tandemly encoding the three genes of the wild-type field or vaccine strains as templates. The amplicons were subjected to HRM analysis using the proprietary High-Resolution Melt software V3.2, which produced derivative melt curves with pre- and post-melt regions (**a**–**c**), aligned melt curves (**d**–**f**), and difference plots (**g**–**i**). The arrowhead and arrow indicate plots of the vaccine (blue lines) and wild-type field genes (red lines), respectively.

Next, we evaluated whether the primer sets could be used for the HRM analysis to discriminate between the wild-type field and vaccine strains using the positive control plasmids tandemly encoding the ORF095, ORF126, and ORF145 genes of the wild-type field strain (tandem field) or the vaccine strain (tandem vaccine). Each primer set could produce amplicons with a single Tm peak for the three genes in the derivative melt plot, but although the peaks between the tandem field and tandem vaccines were very close, they were not identical (Figure 1a). The differences were only about 0.5 °C for each gene (Table 2). However, both the aligned melt curves and the difference plots showed a clear separation between the field and vaccine plots in the three target sites, respectively (Figure 1b,c). It was thus thought that the positive control plasmids (tandem field and tandem vaccine) could be used to produce standard plots for strain classification in clinical specimens.

### 3.3. Analytical Sensitivity of the Assay

**Figure 2:**
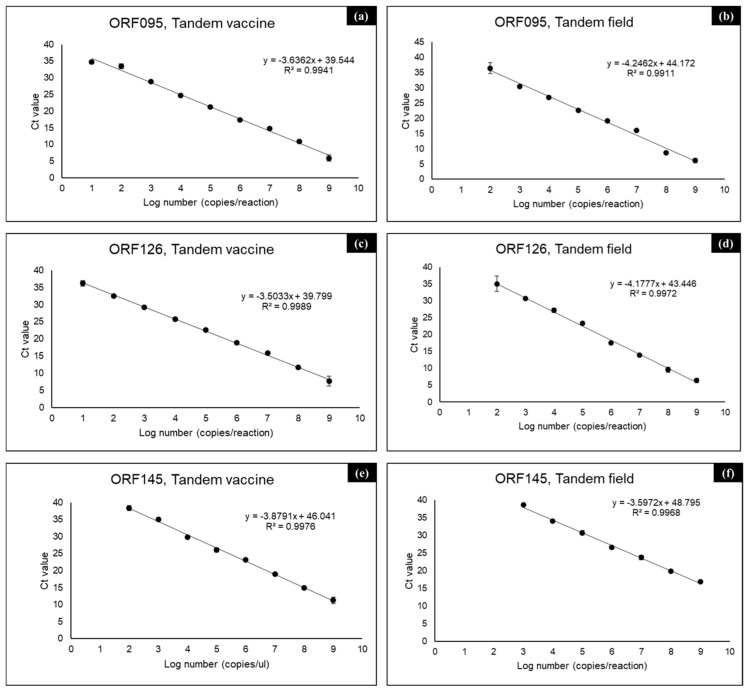
Calibration curves of the real-time PCR results for the ORF095, ORF126, and ORF145 genes for LSDV typing. Each real-time PCR with the primer set for the HRM assay was performed on the QuantStudio 5 system using 10-fold serial dilutions of the control plasmids (tandem field and tandem vaccine) as templates. The mean Ct values obtained at each copy number were plotted to generate the calibration curves, with the bars indicating the standard deviation. The linear regression equation, regression line (dotted line), and coefficient of determination (R^2^) are indicated on each graph. The results for ORF095 (**a**,**b**), ORF126 (**c**,**d**), and ORF145 (**e**,**f**) in the tandem vaccine (**a**,**c**,**e**) or in the tandem field (**b**,**d**,**f**) are shown.

Using 10-fold serial dilutions of the positive control plasmids (field and vaccine), the sensitivity of real-time PCR with the primer set used for HRM typing was determined. Regression lines with high coefficients of determination (R^2^ > 0.99) were obtained from plots of Ct values in all assays (Figure 2). The LODs for targeting the ORF095 and ORF126 genes were both 20 copies per reaction for the vaccine strain and 200 copies per reaction for the wild-type field strain (Table 3). The LODs for targeting the ORF145 gene were 200 and 2000 copies per reaction, respectively, for the vaccine and the wild-type field strains.

### 3.4. Analytical Specificity of the Assay

**Figure 3:**
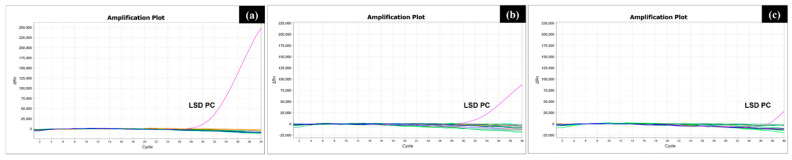
Specificity test of the real-time PCR assay for the ORF095, ORF126, and ORF145 genes for LSDV typing using other bovine viruses. Each real-time PCR with the primer set for the HRM assay was performed on the QuantStudio 5 system using nucleic acids from pseudocowpox virus, bluetongue virus, infectious bovine rhinotracheitis virus, and bovine papillomavirus. The amplification plots for the ORF095 (**a**), ORF126 (**b**), and ORF145 (**c**) genes are shown, with curves indicated by colored lines; pseudocowpox virus (blue), bluetongue virus (green), infectious bovine rhinotracheitis virus (orange), bovine papillomavirus (light green), and recombinant LSDV DNA as a positive control (pink).

The specificities of three primer sets for the ORF095, ORF126, and ORF145 genes were validated using nucleic acids from four bovine viruses (pseudocowpox virus, bluetongue virus, infectious bovine rhinotracheitis virus, and bovine papillomavirus). As shown in Figure 3, no amplification was detected in the real-time PCR for the three genes when nucleic acids from the non-LSDV viruses were used.

### 3.5. Validation of the Assay Using Clinical Samples

**Figure 4:**
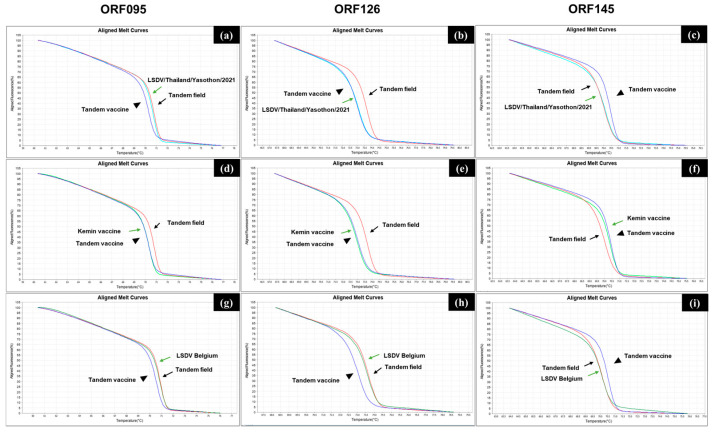
Validation of the HRM assay for LSDV typing using LSDV samples. The assays were performed on the QuantStudio 5 system with the High-Resolution Melt software. The aligned melt curves of the ORF095 (**a**,**d**,**g**), ORF126 (**b**,**e**,**h**), and ORF145 (**c**,**f**,**i**) genes for the Asian recombinant strain in a clinical sample (**a**–**c**), Kemin vaccine strains (**d**–**f**), and the wild-type field strain LSDV/210LSD-249/BUL/16 provided by the Institute in Belgium (LSDV Belgium) (**g–i**) are shown. Black arrows and arrowheads indicate plots of the control DNAs, tandem vaccine, and tandem field, respectively. Green arrows indicate the LSDV samples.

We validated our new assay for the classification of LSDV using the wild-type field strain, vaccine strains, and Asian recombinant strains in clinical specimens. In the assay, the control plasmids (tandem vaccine and tandem field) were used as references for the HRM analysis. The assay results indicated that the aligned melt curves of two vaccine strains, the Kemin strains of the MEVAC live vaccine, matched those of the tandem vaccine plasmid for the three genes (Figure 4d–i). As for the wild-type field strain, the aligned melt curves of the field strain LSDV/210LSD-249/BUL/16 isolated in Bulgaria matched those of the tandem field plasmid for the three genes (Figure 4g–i).

In addition, the HRM assay classified all Asian recombinant strains (already confirmed) in 15 skin nodule samples collected from eight provinces of Thailand as the 2.5 recombinant strain. Representative results are shown in Figure S2a–c, in which the aligned melt curves of the ORF095 and ORF145 genes of the recombinant strain matched those of the reference for the field strain, and the curve of the ORF126 gene of the strain matched that of the reference for the vaccine strain. Results from seven other collection sites are also shown in Figure S2. It should be noted that several Tm values obtained from the assays for recombinant strains showed intermediate values between the values of the field and vaccine strains’ references, which made classification difficult (Table S1).

## 4. Discussion

A live-attenuated vaccine for LSD is commercially available and is effective in protecting cattle against LSDV infection, but a strategy for the differentiation of infected from vaccinated animals (DIVA) should be included to control the disease using the live vaccine because it causes the Neethling response side effects [33,34,35,36,37]. However, the recently emerged recombinant strain, a hybrid of the wild-type field and vaccine strains, has spread widely in East and Southeast Asia [17,18,19,20,21,22,23], which interferes with DIVA because the DNA regions derived from the wild-type field strain are widely and mosaically distributed in the recombinant virus genome, where the vaccine strain genome is the backbone [54,55]. Given this situation, a simple method for differentiating the recombinant strains is also needed. DNA genome sequencing (Sanger method and next-generation sequencing) is a common and reliable method used for LSDV genotyping and molecular epidemiology [56,57,58], but it is a laborious, time-consuming, and expensive process. PCR followed by a restriction fragment length polymorphism (RFLP) assay is a simple method to identify nucleotide substitution(s), characterizing strains by restriction enzyme digestion, and there is one report of differentiation between LSDV and SPPV [59]. However, this method requires researchers to find a suitable restriction site and to perform gel electrophoresis, which increases the risk of cross-contamination. PCR followed by amplicon size separation using gel electrophoresis can be available for differentiation if there is a large insertion/deletion that characterizes strains. However, as with the RFLP assay, there is a risk of contamination. Moreover, SNPs are difficult to detect.

Recently, real-time PCR has been utilized for the detection of insertions/deletions or SNPs capable of viral genotyping, and there are reports of a differentiation method based on real-time PCR for LSDV, as described below. For example, one real-time PCR-based assay comprises a closed-tube and single-step method, thereby reducing the risk of contamination and improving the assay throughput. Moreover, it is also highly sensitive and is therefore considered a cost-effective method for a pathogen genotyping [42]. The real-time PCR assay is generally classified into two methods that use a sequence-specific oligonucleotide with a hydrolysis fluorescent probe (TaqMan probe) or a fluorescent dye intercalating into double-stranded DNA. The TaqMan probe method allows for a multiplex assay by using multiple color probes, but the probes are expensive. On the other hand, real-time PCR using an intercalating fluorescent dye allows for genotyping based on the difference in Tm values calculated from the melting curve analysis after a PCR run. Furthermore, HRM analysis using a saturating dye (e.g., LCGreen, SYTO 9, or EVAGreen) improves accuracy and resolution to discriminate genetic variants [42]. Gene typing using melting analysis can be performed at a low cost, but multiple wells are required for a multiplex assay.

To date, several reports have been published on a real-time PCR-based assays capable of DIVA for LSDV, but no assays are available to differentiate the wild-type field, Asian recombinant, and vaccine strains. Agianniotaki et al. (2017) developed a Taqman probe-based qPCR method targeting the ORF011 (GPCR) gene for a DIVA assay [60]. Pestova et al. (2018) combined an HRM assay with real-time PCR using EvaGreen dye targeting ORF010 (LAP/PHD-finger protein) to differentiate SPPV, GTPV, and vaccine and field strains of LSDV [48]. Chibssa et al. (2019) reported that the capripoxvirus homolog of the variola virus B22R gene can be used in an HRM assay to differentiate the field and vaccine strains of SPPV, GTPV, and LSDV [49]. Kumar et al. (2023) used the HRM assay using SYBR green dye targeting the inverted terminal repeat (ITR) region for DIVA between the Indian strain vaccine (Lumpi-Provacind) P50 and wild-type field strains [51]. However, these assays cannot identify recombinant strains because only one gene region was used for differentiation. Recently, a probe-based duplex real-time PCR method for DIVA in LSDV was reported [61]. This assay targets the ORF133 (DNA-ligase-like protein) and ORF144 (Kelch-like protein) genes for specific detection of the wild-type field and vaccine strains, respectively, but cannot differentiate the wild-type field and recombinant strains because recombinant strains produce the same result as the wild-type field strain. If this method were to be used to distinguish between the three types of LSDV, an assay targeting vaccine strains would also be needed to detect recombinant strains.

We considered that multi-locus sequence typing (MLST), which is commonly used for a bacterial genotyping [62], is required for differentiation between the wild-type field, recombinant, and vaccine strains of LSDV. As an example, HRM-based MLST assays have been developed for pathogenic bacteria and viruses [63,64,65,66] and provide better resolution, especially for the analysis of recombinant species [42]. Therefore, we selected three genes (ORF095, ORF126, and ORF145) for differentiation using the multi-locus HRM assay. We rejected the use of TaqMan probes because they are expensive; at least four probes for two loci must be required for differentiation. For target gene selection, we targeted only the cluster 2.5 recombinant virus, which, among the five clusters 2.1–2.5, is the only strain spreading throughout East and Southeast Asia [14]. The ORF126 gene encodes EEV glycoprotein, reported to have a 27 bp deletion in the vaccine strains [47,67], a deletion also found in the recombinant strains. The ORF095 and ORF145 genes have not been used as a diagnostic tool until now. The ORF095 gene encodes a virion core protein, which is conserved between the wild-type field and recombinant strains but not in the vaccine strains, which have a 48 bp deletion in the amplified region. The ORF145 gene encodes an ankyrin repeat protein that differs only in the vaccine strains, which have a total of 72 bp insertions and many nucleotide substitutions. Our results indicate that the multi-locus HRM assay using the three genes enables us to clearly differentiate between the wild-type field, vaccine, and recombinant strains of LSDV. The HRM assay with either combination of ORF126 and ORF095 or ORF145 genes is considered sufficient for the differentiation of LSDV.

However, it should be noted that the LOD for the ORF145 gene of the wild-type field strain was especially low (2000 copies/reaction). One of the possibilities is due to primer mismatch. The ORF145 gene of the wild-type field strains has one mismatch with the forward primer at the fifth nucleotide and also one with the reverse primer, whereas that of the vaccine strains has a mismatch with the forward primer at the second nucleotide from the last but no mismatch with the reverse primer (Table 1 and Figure S1). Therefore, the primer set will be improved in future work. Moreover, SPPV and GTPV were not evaluated in the HRM assay, although it is possible that these viruses can be amplified by real-time PCR based on the primer sequences (Figure S1). Therefore, it may be necessary to combine our assay with another HRM assay that can differentiate between capripoxviruses [49] or to improve the primer set(s) to produce negative results in SPPV and GTPV samples.

In this study, we used MeltDoctor HRM Master Mix with the saturated intercalating dye SYTO 9. The PCR amplicons derived from both the tandem vaccine and tandem field plasmids exhibited only small differences in their Tm values, approximately 0.41–0.68 °C on average for the three genes. Moreover, some recombinant virus strains showed intermediate Tm values, which might be attributed to nucleotide substitution(s) in the target region. Nonetheless, the melt curves were clearly separated and correctly displayed in the aligned melt curve plot, suggesting that the HRM assay should not be replaced with the standard melt curve analysis.

To the best of our knowledge, this is the first report of an HRM assay that enables differentiation between three groups of LSDV, namely, the wild-type field, vaccine, and Asian recombinant (cluster 2.5) strains. This cost-effective assay can be used not only for the rapid detection of LSDV in clinical samples from cattle but also for rapid differentiation between an outbreak case caused by the wild-type field and recombinant strains and Neethling reactions from a live-attenuated vaccine. Although the feasibility and concept of adopting a multi-locus HRM assay for LSDV differentiation has been proven, this study has some other limitations that should be addressed in the future work. The size and source of samples tested were limited in this study, and they need to be expanded for generalization. It should also be noted that the compatibility of the assay with PCR platforms other than the QuantStudio 5 system needs to be verified for widespread use. Further, this assay should be revised if a new recombinant virus strain emerges or a novel live-attenuated vaccine is developed. If this revision is not required, whole-genome sequencing using the Oxford Nanopore platform with tiled PCR amplicons will be a rapid and more economical method [68].

## 5. Conclusions

The DIVA concept is important for controlling viral diseases in livestock with a vaccine. For LSDV, the emergence and spread of the recombinant strain, a chimera of the wild-type field and vaccine strains, makes DIVA difficult. This study provides a novel, rapid, and cost-effective method based on a multi-locus (ORF095, ORF126, and ORF145 genes) HRM assay that enable differentiation between the wild-type field, vaccine, and recombinant strains of LSDV. We believe that this novel diagnostic tool will be useful to control LSD in cattle in many regions where a real-time PCR assay can be routinely performed.

## Figures and Tables

**Table 1:** Primer sequences for real-time PCR with an HRM assay for the differentiation of LSDV strains.

Primer Name	Primer Sequence	Amplicon Size (bp) of Wild-Type or Vaccine Strains
LSD 095-Fw	5′-TACTAGATTCCATTTATATTCGAAG-3′	180 or 131
LSD 095-Rv	5′-GAGATTTAATATAGACAAACTGGATGA-3′	
LSD 126-Fw	5′-TAGAAAATGGATGTACCACA-3′	133 or 106
LSD 126-Rv	5′-AATCGTTGTTACAACTCAAA-3′	
LSD 145-Fw	5′-AACTGTATGTCGTTAAGGG-3′	127 or 199
LSD 145-Rv	5′-CATAAAAAGATAACACTTAACACT-3′	

**Table 2:** Comparison of the peak Tm values between the wild-type field and vaccine strain genes in each HRM assay.

Template	ORF095		ORF126		ORF145	
	Tm Value	*t*-Test	Tm Value	*t*-Test	Tm Value	*t*-Test
Tandem field	70.94 ± 0.02	*p* < 0.0001	73.54 ± 0.01	*p* < 0.0001	70.10 ± 0.01	*p* < 0.0001
Tandem vaccine	70.45 ± 0.01		73.10 ± 0.01		70.58 ± 0.01	

Mean Tm values (°C) were calculated from triplicate reactions and expressed as mean ± standard deviation. Tm values were evaluated using Student’s *t*-test, where a *p* value less than 0.05 indicated a significant difference.

**Table 3:** LODs of each real-time PCR assay.

Template	ORF095		ORF126		ORF145	
	LOD	Ct	LOD	Ct	LOD	Ct
Tandem field	200	36.46 ± 1.85	200	35.00 ± 2.28	2000	38.63 ± 0.23
Tandem vaccine	20	34.72 ± 0.53	20	36.24 ± 0.89	200	38.38 ± 0.70

LOD is expressed as copies/reaction, and Ct values at the endpoint are expressed as mean ± standard deviation.

## Data Availability

No original data were stored in a public repository. The data that support the study findings are available upon request.

## Supplementary Materials

The following supporting information can be downloaded at: https://www.mdpi.com/article/10.3390/vetsci12030213/s1, Figure S1: Multiple alignment of the target regions used for the multi-locus real-time PCR and high resolution melting assay for LSDV typing; Figure S2: Representative results of the HRM assay for the typing of LSDV clinical samples collected from eight provinces in Thailand; Table S1: Tm values from the melt curve analysis for LSDV typing.

## Abbreviations

The following abbreviations are used in this manuscript:

LSD	Lumpy skin disease
DIVA	Differentiation of infected from vaccinated animals
HRM	High-resolution melting
WOAH	World Organization for Animal Health
LSDV	Lumpy skin disease virus
SPPV	Sheeppox virus
GTPV	Goatpox virus
SNP	Single nucleotide polymorphism
NIAH	National Institute of Animal Health
LOD	Limit of detection
RFLP	Restriction fragment length polymorphism
MLST	Multi-locus sequence typing
