# Supplementary material for: Development of a Multi-Locus Real-Time PCR with a High-Resolution Melting Assay to Differentiate Wild-Type, Asian Recombinant, and Vaccine Strains of Lumpy Skin Disease Virus
Source: Vet Sci. 2025 Mar 1;12(3):213. doi: 10.3390/vetsci12030213 (PMC11945404; doi:10.3390/vetsci12030213)
Supplement: Supplementary file 1 [file vetsci-12-00213-s001.zip › vetsci-3480846-supplementary.pdf]

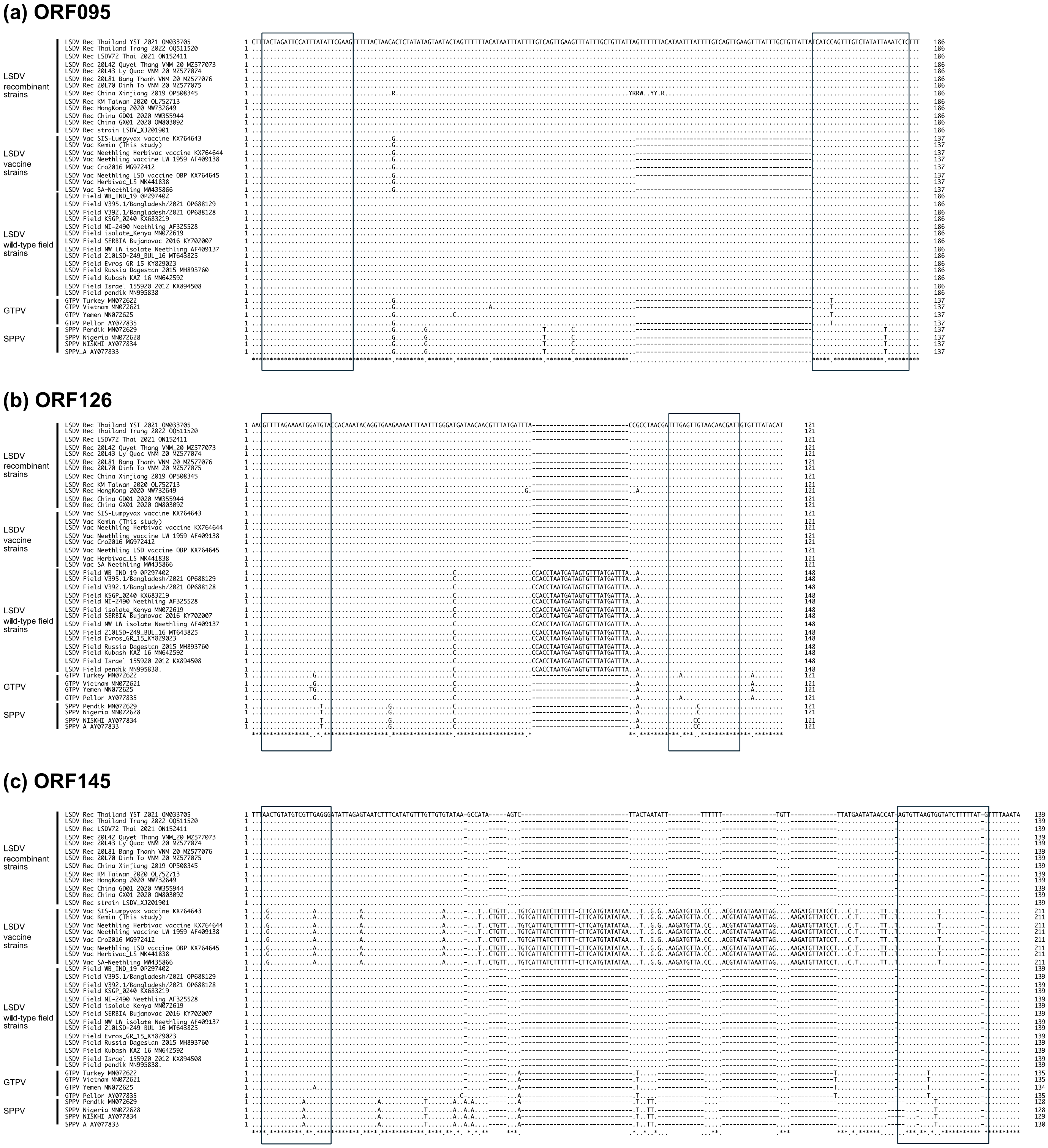

Figure S1. Multiple alignment of the target regions used for the multi-locus real-time PCR and high resolution melting assay for LSDV typing.

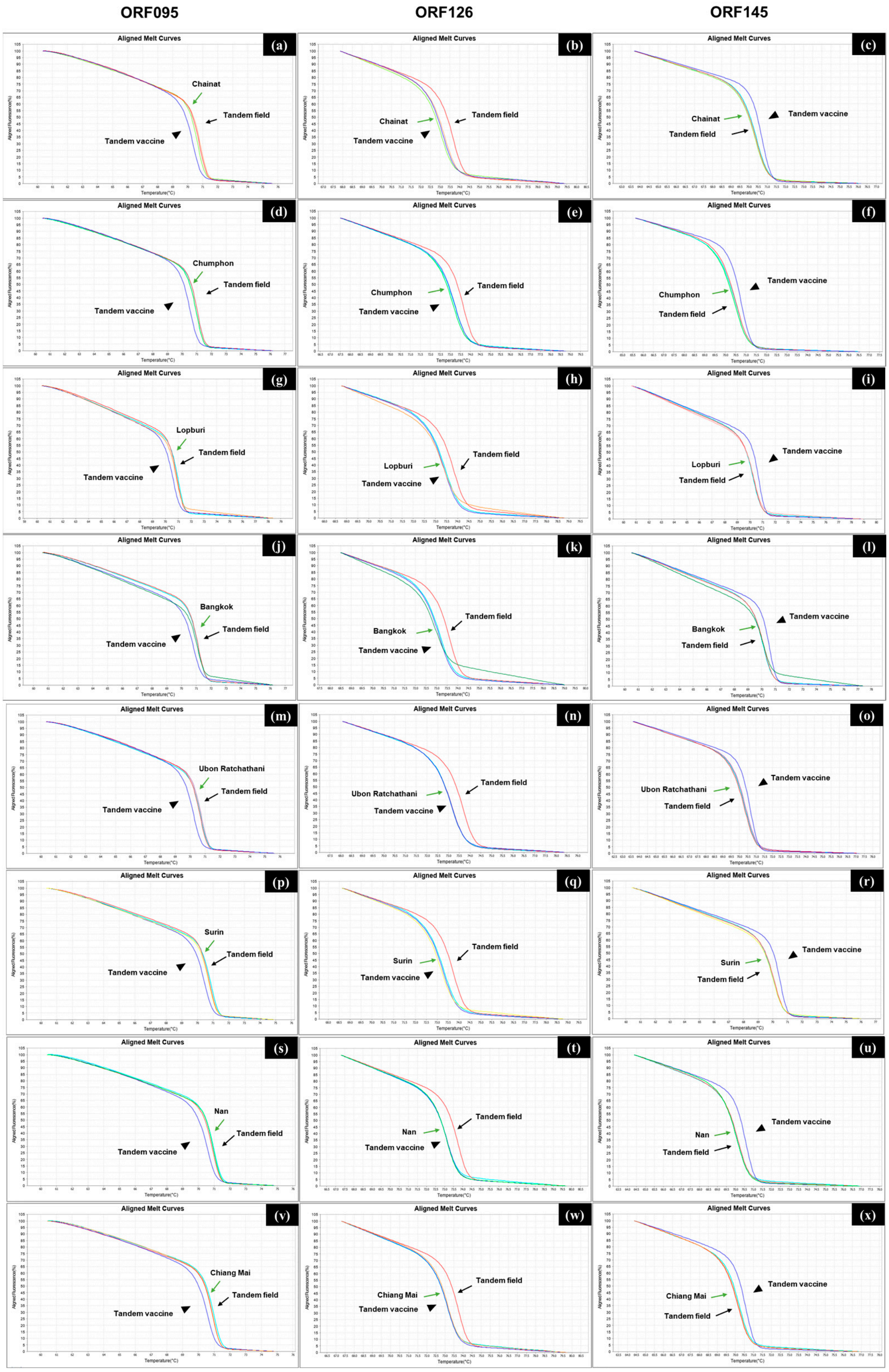

Supplementary Figure S2. Representative results of the HRM assay for the LSDV typing of the LSDV clinical samples collected from eight provinces in Thailand. The assays were performed on the QuantStudio 5 system with the High-Resolution Melt software. The align melt curves of the ORF095 (a, d, g, j, m, p, s, and v), ORF126 (b, e, h, k, n, q, t, and w), and ORF145 (c, f, i, l, o, r, u, and x) genes for the Asian recombinant strains in LSDV clinical samples collected in Chainat (a–c), Chumpon (d–f), Lopburi (g–i), Bangkok (j–l), Ubon Ratchathani (m–o), Surin (p–r), Nan (s–u), and Chiang Mai (v–x). Black arrows and arrowheads indicate plots of the control DNAs for tandem vaccine and tandem field, respectively. Green arrows indicate the LSDV samples.

Supplementary Table S1. Tm values from the melt curve analysis for LSDV typing.

DNA template	ORF095	ORF126	ORF145
	**Tm value**	**Tm value**	**Tm value**
**Control plasmids**			
Tandem vaccine (n = 9)	70.46 ± 0.06	73.10 ± 0.05	70.58 ± 0.04
Tandem field (n = 9)	70.92 ± 0.10	73.52 ± 0.16	70.01 ± 0.32
**LSDV vaccine strains**			
Kemin strain (n = 3)	70.46 ± 0.08	73.19 ± 0.06	70.41 ± 0.03
**LSDV wide-type strains**			
LSDV/210LSD-249/BUL/16 (n = 6)	70.83 ± 0.08	73.48 ± 0.04	70.05 ± 0.05
**LSDV fecombinant strains**			
Yasothon (n = 3)	71.02 ± 0.08	73.29 ± 0.11	70.13 ± 0.10
Ubon 66A19049 (n = 3)	70.83 ± 0.01	73.11 ± 0.10	70.34 ± 0.13
Chumphon 67A02130 (SK1) (n = 1)	70.81	73.41	70.68
Chumphon 67A02130 (SK2) (n = 1)	70.92	73.13	70.41
Chumphon 67A02130 (NS1) (n = 1)	70.79	73.16	70.47
Chainat 66A17956 (n = 1)	70.64	73.09	70.34
Lopburi 64A138156 P.7 (n = 1)	70.91	73.25	70.23
Lopburi 65A11171 P.7 (n = 1)	70.97	73.30	70.35
Lopburi 65A01032 P.7 (n = 1)	70.77	73.29	70.32
Surin 66A17785 (n = 1)	70.82	73.15	70.17
Surin 66A17786 (n = 1)	70.88	73.23	70.22
Bangkok 66A18735 (n = 1)	70.84	73.36	70.21
Chiangmai 509800/64 (n = 1)	70.90	73.18	70.24
Nan 504831/64 (n = 1)	70.98	73.31	70.27
Roi Et 991.2 (n = 1)	71.08	73.33	70.62

Replication is indicated as n = x, where x is the number of replicates.

Tm values obtained from multiple replicates are expressed as means ± standard deviations.
